# Inhibition of BACE1 affected both its Aβ producing and degrading activities and increased Aβ42 and Aβ40 levels at high-level BACE1 expression

**DOI:** 10.1016/j.jbc.2024.107510

**Published:** 2024-06-27

**Authors:** Irem Ulku, Rocher Leung, Fritz Herre, Lina Walther, Adeola Shobo, Paul Saftig, Mark A. Hancock, Filip Liebsch, Gerhard Multhaup

**Affiliations:** 1 Department of Pharmacology and Therapeutics, McGill University, Montreal, Quebec, Canada; 2 Integrated Program in Neuroscience, McGill University, Montreal, Quebec, Canada; 3 Biochemisches Institut, CAU Kiel, Kiel, Germany; 4 Department of Chemistry, Institute of Biochemistry, University of Cologne, Cologne, Germany

**Keywords:** Alzheimer’s disease, amyloid production, amyloid clearance, BACE1 amyloidolytic activity, secretase inhibitors

## Abstract

The beta-site amyloid precursor protein cleaving enzyme 1 (BACE1) is the predominant β-secretase, cleaving the amyloid precursor protein (APP) *via* the amyloidogenic pathway. In addition, BACE1 as an amyloid degrading enzyme (ADE), cleaves Aβ to produce the C-terminally truncated non-toxic Aβ fragment Aβ34 which is an indicator of amyloid clearance. Here, we analyzed the effects of BACE1 inhibitors on its opposing enzymatic functions, *i.e.*, amyloidogenic (Aβ producing) and amyloidolytic (Aβ degrading) activities, using cell culture models with varying BACE1/APP ratios. Under high-level BACE1 expression, low-dose inhibition unexpectedly yielded a two-fold increase in Aβ42 and Aβ40 levels. The concomitant decrease in Aβ34 and secreted APPβ levels suggested that the elevated Aβ42 and Aβ40 levels were due to the attenuated Aβ degrading activity of BACE1. Notably, the amyloidolytic activity of BACE1 was impeded at lower BACE1 inhibitor concentrations compared to its amyloidogenic activity, thereby suggesting that the Aβ degrading activity of BACE1 was more sensitive to inhibition than its Aβ producing activity. Under endogenous BACE1 and APP levels, “low-dose” BACE1 inhibition affected both the Aβ producing and degrading activities of BACE1, *i.e.*, significantly increased Aβ42/Aβ40 ratio and decreased Aβ34 levels, respectively. Further, we incubated recombinant BACE1 with synthetic Aβ peptides and found that BACE1 has a higher affinity for Aβ substrates over APP. In summary, our results suggest that stimulating BACE1’s ADE activity and halting Aβ production without decreasing Aβ clearance could still be a promising therapeutic approach with new, yet to be developed, BACE1 modulators.

Alzheimer's disease (AD) is a chronic, biologically heterogeneous neurodegenerative brain disorder that is neuropathologically characterized by senile plaques composed of amyloid-β (Aβ) peptides as well as neurofibrillary tangles consisting of hyperphosphorylated tau (1). Two major enzymes within the “amyloidogenic” pathway, the β-site amyloid precursor protein cleaving enzyme-1 (termed β-secretase or BACE1) and the γ-secretase complex, drive the conversion of the amyloid precursor protein (APP) to the clinically relevant Aβ40 and Aβ42 species in AD (2). To initiate the production of Aβ peptides, BACE1 cleaves cellular APP (starting with Asp1 of the Aβ sequence) and releases the soluble APPβ (sAPPβ) ectodomain (3, 4, 5, 6, 7).

BACE1 is the only amyloid-degrading enzyme (ADE) that has a major role in Aβ production and degradation (8, 9, 10, 11, 12, 13). Within the “amyloidolytic” pathway, soluble forms of Aβ are cleaved by BACE1 into non-toxic and non-aggregating Aβ34 which we previously discovered as an indicator of amyloid clearance in amyloid-positive individuals with mild cognitive impairment who later progressed to dementia (10, 11, 14).

The attractiveness of BACE1 as a therapeutic target has been supported (i) by the natural protective Icelandic APP-A673T genetic mutation that partially prevents the initial amyloidogenic cleavage and yielded ∼30% lower levels of Aβ40 and Aβ42 (15, 16) and (ii) by BACE1 inhibitors that successfully decreased Aβ40 and Aβ42 levels to a similar degree *in vivo* (see review (17)).

While BACE1 inhibitors have been thoroughly investigated as candidate therapeutics during the symptomatic and/or pre-symptomatic stages in patients with AD, all such clinical trials were halted due to a lack of efficacy and/or safety concerns (18, 19). Recent *in vitro* studies, however, have reexamined BACE1 under so-called “low-dose BACE1 inhibitor” conditions, as defined by inhibitor concentrations that prompt <50% decreases in Aβ secretion without affecting synaptic transmission (20). Since the partial reductions in Aβ production could mimic the putative protective effect of the Icelandic mutation (APP A673T), such studies have proposed that asymptomatic populations may benefit from the long-term administration of low-dose BACE1 inhibitors to prevent AD by ideally yielding 30% reduced Aβ production (21). At the same time, renewed efforts to develop more selective, less toxic BACE1 inhibitors are emerging (22, 23, 24, 25).

Given that BACE1 can mediate opposing amyloidogenic and amyloidolytic enzymatic activities, *that is,* Aβ production *versus* Aβ clearance, respectively, BACE1 inhibitors that inadvertently reduce amyloid clearance may complicate their intended use to attenuate amyloid production (10). While promoting Aβ clearance by increasing BACE1 expression would not be considered a therapeutic approach because of its β-secretase activity, BACE1 modulators that address the dual mechanism of action could improve Aβ cleavage whilst halting Aβ production. Increased protein levels and enzymatic activity of BACE1 in AD brain and blood may occur to compensate for the decreased clearance in the AD phenotype (26, 27, 28, 29, 30). Therefore, the current study was specifically designed to examine how disease-modifying secretase therapeutics, BACE1 inhibitors *versus* γ-secretase modulators affect Aβ homeostasis in cell culture models with varying APP and BACE1 expression levels.

## Results

### *In vitro* model #1—inhibition of BACE1 activity in wild-type SH-SY5Y cells

**Figure 1:**
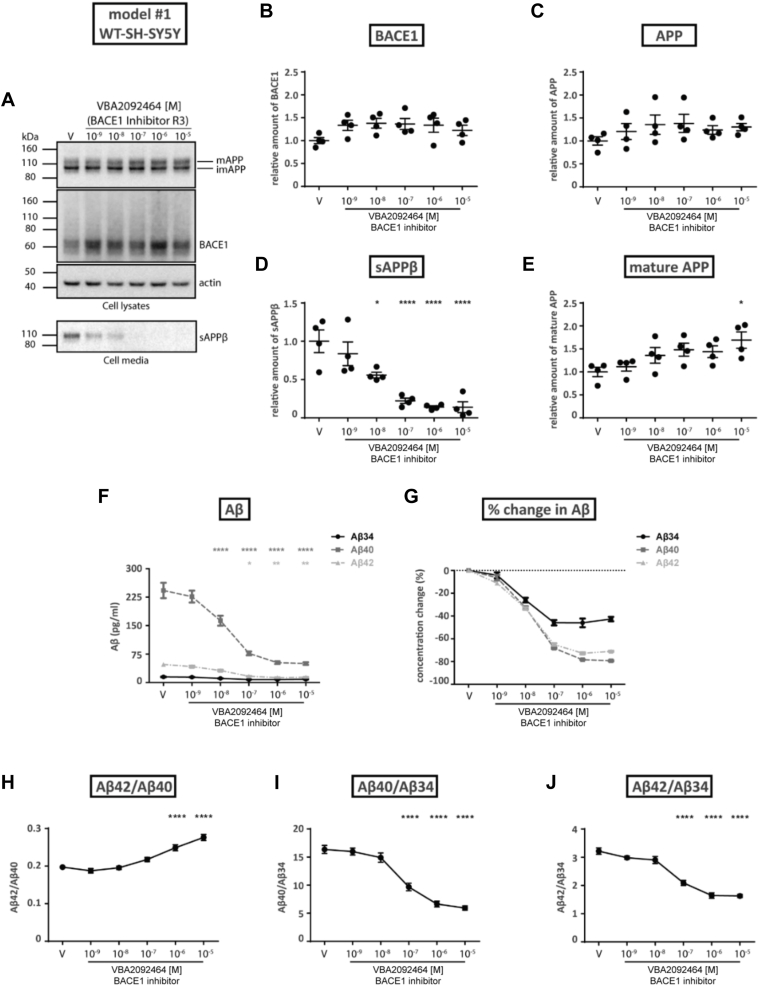
**Effects of VBA2092464 (R3) BACE1 inhibitor on *human SH-SY5Y cells*.** Wild-type SH-SY5Y cells were treated with vehicle (V) or varying concentrations of the VBA2092464 (R3) BACE1 inhibitor. *A*, Western blotting (representative of 4 independent experiments) of cell lysates for the detection of APP (upper band, mature; lower band, immature), BACE1, and actin (control), or cell media for secreted sAPPβ. *B–E*, conversion of WB data to relative amounts of BACE1, APP, sAPPβ, or mature APP as a function of BACE1 inhibitor concentration. *F*, quantification of Aβ34, Aβ40, and Aβ42 levels in cell media as a function of BACE1 inhibitor concentration as determined by MSD immunoassay. *G*, conversion of MSD data (pg/ml) to the percentage change of Aβ levels (relative to the vehicle condition). *H–J*, conversion of MSD data (pg/ml) to specified Aβ ratios. Statistics: Bars and error bars indicate mean ± s.e.m. Dunnet’s *post hoc* tests were performed for pairwise comparisons; selected comparisons are highlighted ∗∗∗∗*p* < 0.0001, ∗∗∗*p* < 0.001, ∗∗*p* < 0.01, ∗*p* < 0.05. *B*, BACE1, 1-WAY ANOVA, F(5,18) = 1.590, *p* = 0.2133, (*C*) APP, 1-WAY ANOVA, F(5,18) = 0.8343, *p* = 0.5423, (*D*) sAPPβ, 1-WAY ANOVA, F(5,18) = 15.68, *p* < 0.0001, (*E*) mature APP, 1-WAY ANOVA, F(5,18) = 3.315, *p* < 0.05, (*F*) Absolute Aβ, 2-WAY ANOVA, interaction F(10,54) = 41.65, *p* < 0.0001, row factor F(5,54) = 74.57, *p* < 0.0001, column factor F(2,54) = 549.2, *p* < 0.0001, (*H*) Aβ42/Aβ40, 1-WAY ANOVA, F(5,18) = 33.71, *p* < 0.0001, (*I*) Aβ40/Aβ34, 1-WAY ANOVA, F(5,18) = 56.02, *p* < 0.0001, (*J*) Aβ42/Aβ34, 1-WAY ANOVA, F(5,18) = 58.38, *p* < 0.0001.

To investigate how pharmacological inhibition can affect endogenous BACE1 amyloidogenic and amyloidolytic activities in wild-type human neuroblastoma SH-SY5Y cells (Fig. 1), we measured AD-related protein levels in the absence or presence of the BACE1-selective inhibitor VBA2092464 (termed “R3”). Expression levels in cell lysates or media were analyzed by Western blotting (Fig. 1*A*) to determine the relative amounts of BACE1 (Fig. 1*B*), total APP (Fig. 1*C*), sAPPβ (Fig. 1*D*), and mature APP (Fig. 1*E*). Indicative of reduced BACE1-mediated cleavage of APP (*i.e.*, reduced amyloidogenic activity), sAPPβ levels were significantly decreased with increasing inhibitor concentration (Fig. 1*D*, IC50 = 10^−8^ M). Our data agrees with the published IC50 for VBA2092464 (R3) in the nanomolar range (30 nM for human recombinant BACE1 with an APP-based substrate peptide (31)). At inhibitor concentrations of 10^−8^ M and higher, BACE1 (Fig. 1*B*) and total APP (Fig. 1*C*) levels remained unaltered compared to controls, whereas mature APP increased by ∼1.5-fold (Fig. 1*E*).

The supernatants of wild-type SH-SY5Y cells were also analyzed for Aβ levels using our previously established multiplex MSD assay (10). The quantification of Aβ34, Aβ40, and Aβ42 (Fig. 1, *F* and *G*) revealed an approximately 30% decrease for all three species at an inhibitor concentration of 10^-8^ M. The relative change in Aβ34 plateaued at a lower VBA2092464 (R3) concentration of 10^−7^ M (∼45%) compared to Aβ40 or Aβ42 at 10^−6^ M (∼80% for Aβ40; ∼70% for Aβ42). These findings imply that VBA2092464 (R3) had a saturating effect on the amyloidolytic activity of BACE1 at lower inhibitor concentrations as compared to its amyloidogenic activity. Importantly, the ratio of Aβ42/Aβ40 was significantly increased at inhibitor concentrations above 10^−7^ M (Fig. 1*H*), whereas the ratios Aβ40/Aβ34 (Fig. 1*I*) or Aβ42/Aβ34 (Fig. 1*J*) were significantly decreased under identical conditions. Notably, the observed 0.2 to 0.3 increase in the Aβ42/Aβ40 ratio for wild-type SY5Y cell supernatant is significantly higher compared to the ratio in cerebrospinal fluid (CSF) of non-demented controls (0.08) and patients with mild cognitive impairment (MCI)/AD (0.04) (32) which points towards a biased inhibition of Aβ42 degradation in the presence of the VBA2092464 (R3) inhibitor.

### *In vitro* model #2—inhibition of BACE1 activity in stable BACE1-overexpressing SH-SY5Y cells

To examine BACE1 inhibition under conditions of high BACE1 expression, we tested stable BACE1-overexpressing SH-SY5Y cells (BACE1-SH-SY5Y) in the absence or presence of a wider array of BACE1 inhibitors (see Experimental procedures): VBA2092464 (R3) as well as four oxazine/amidine-type compounds with different acid dissociation constants (N6: pKa = 9.5; N8: pKa = 7.7; N10: pKa = 7.0; N11: pKa = 6.4) to test if BACE1 inhibition is pH dependent.

**Figure 2:**
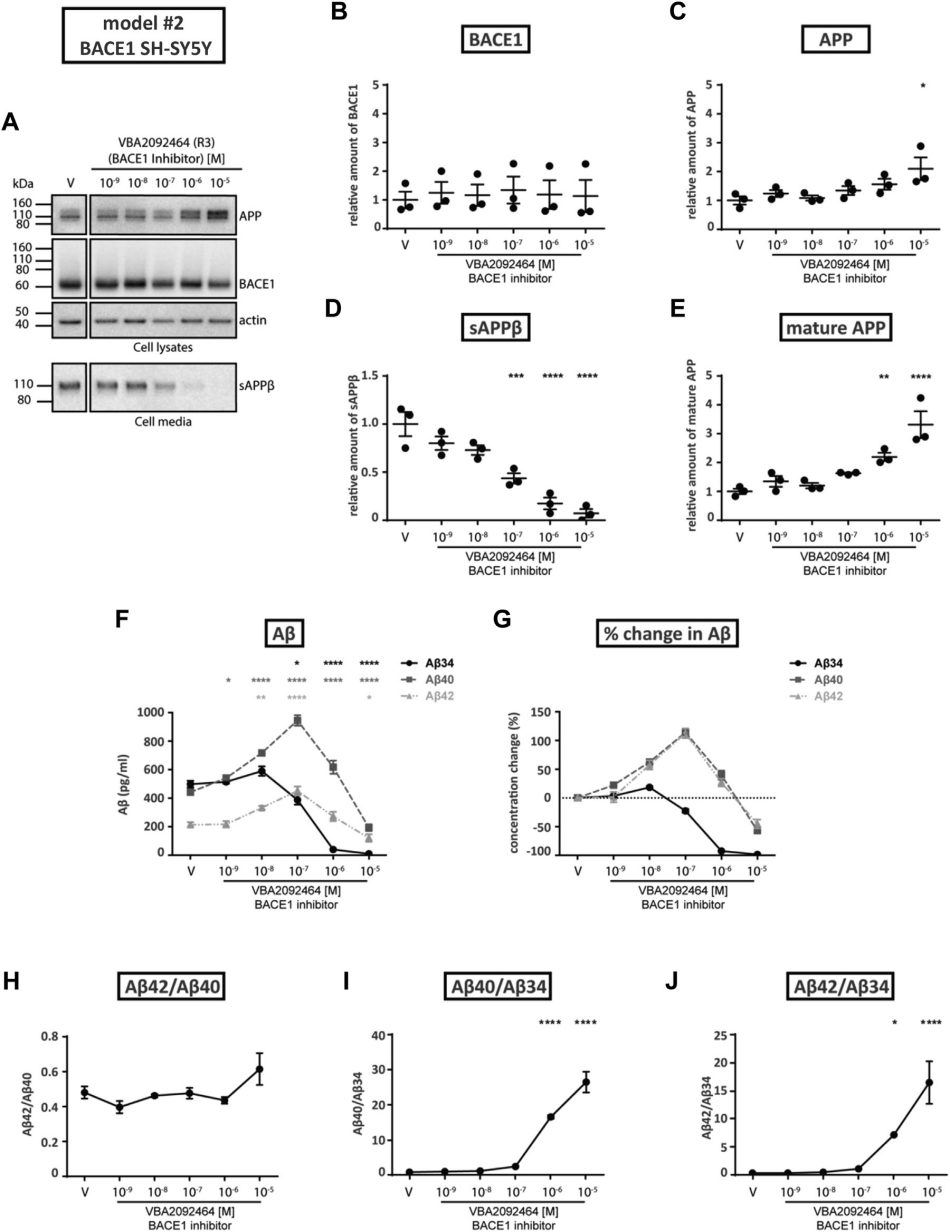
**Effects of VBA2092464 (R3) BACE1 inhibitor on *stably BACE1 overexpressing SH-SY5Y cells*.** BACE1-SH-SY5Y cells were treated with vehicle (V) or varying concentrations of the VBA2092464 (R3) BACE1 inhibitor. *A*, Western blotting (representative of three independent experiments) of cell lysates for the detection of APP (*upper band*, mature; *lower band*, immature), BACE1, and actin (control), or cell media for secreted sAPPβ. *B–E*, conversion of WB data to relative amounts of BACE1, APP, sAPPβ, or mature APP as a function of BACE1 inhibitor concentration. *F*, quantification of Aβ34, Aβ40 and Aβ42 levels in cell media as a function of BACE1 inhibitor concentration as determined by ELISA and MSD (Aβ34). *G*, conversion of data from Figure 2*F* (pg/ml) to percentage change of Aβ levels (relative to the vehicle condition). *H–J*, conversion of data from Figure 2*F* (pg/ml) to specified Aβ ratios. Statistics: Bars and error bars indicate mean ± s.e.m. Dunnet’s *post hoc* tests were performed for pairwise comparisons; selected comparisons are highlighted ∗∗∗∗*p* < 0.0001, ∗∗∗*p* < 0.001, ∗∗*p* < 0.01, ∗*p* < 0.05. *B*, BACE1, 1-WAY ANOVA, F(5,12) = 0.06869, *p* = 0.9959, (*C*) APP, 1-WAY ANOVA, F(5,12) = 3.730, *p* < 0.05, (*D*) sAPPβ, 1-WAY ANOVA, F(5,12) = 25.71, *p* < 0.0001, (*E*) mature APP, 1-WAY ANOVA, F(5,12) = 15.25, *p* < 0.0001, (*F*) Absolute Aβ, 2-WAY ANOVA, interaction F(10,36) = 39.26, *p* < 0.0001, row factor F(5,36) = 146.4, *p* < 0.0001, column factor F(2,36) = 247.4, *p* < 0.0001, (*H*) Aβ42/Aβ40, 1-WAY ANOVA, F(5,12) = 2.714, *p* = 0.0727, (*I*) Aβ40/Aβ34, 1-WAY ANOVA, F(5,12) = 78.10, *p* < 0.0001, (*J*) Aβ42/Aβ34, 1-WAY ANOVA, F(5,12) = 17.73, *p* < 0.0001.

When overexpressing BACE1-SH-SY5Y cells were treated with increasing VBA2092464 (R3) concentrations, Western blotting (Fig. 2*A*) revealed that BACE1 levels (Fig. 2*B*) remained unaltered across all inhibitor concentrations in contrast to the altered levels of total APP (Fig. 2*C*; unaltered at 10^−6^ M, but two-fold increase at 10^−5^ M) due to altered full-length mature APP (Fig. 2*E*; unaltered at 10^−7^ M, but more than three-fold increase at 10^−5^ M). Notably, the significant decrease in sAPPβ levels was half-maximal (IC50) at 10^−7^ M VBA2092464 (R3), and maximal inhibition (∼85%) was observed starting at 10^−6^ M (Fig. 2*D*). Under identical assay conditions, all other BACE1 inhibitors tested (N6, N8, N10, N11) yielded similar outcomes to VBA2092464 (R3) in terms of protein expression (Supporting information S3). Overall, the BACE1-SH-SY5Y cells exhibited consistent changes in sAPPβ (decreased levels), mature APP (increased levels), and BACE1 (unaltered levels) across all six BACE1 inhibitors tested.

**Figure 3:**
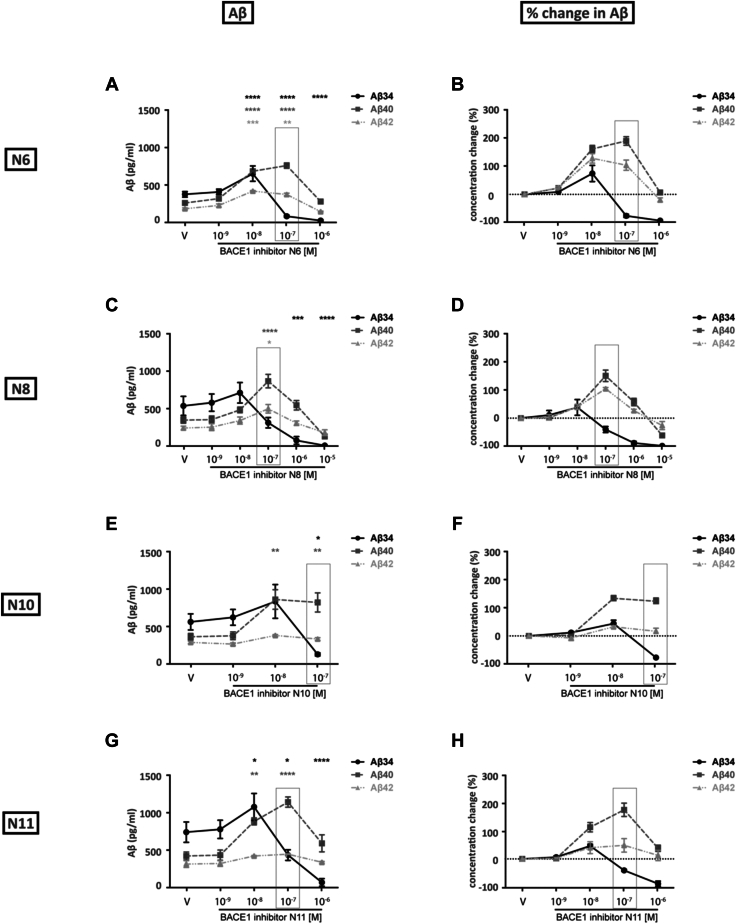
**Effect of inhibitors with different pKa values on *stably BACE1 overexpressing SH-SY5Y cells*.** BACE1-SH-SY5Y cells were treated with vehicle (V) or varying concentrations of the N6, N8, N10 or N11 BACE1 inhibitor. Three independent experiments were performed. Due to the different toxicities of inhibitors, the concentrations covered are different. The concentration of 10^−7^ M for all inhibitors is marked with a box in the figure to allow an easier comparison. *A, C, E*, and *G*, quantification of Aβ34, Aβ40 and Aβ42 levels in cell media as a function of BACE1 inhibitor concentration as determined by ELISA and MSD (Aβ34). *B, D, F*, and *H*, conversion of data from absolute amounts (pg/ml) to the percentage change of Aβ levels (relative to the vehicle condition). Statistics: Bars and error bars indicate mean ± s.e.m. Dunnet’s *post hoc* tests were performed for pairwise comparisons; selected comparisons are highlighted ∗∗∗∗*p* < 0.0001, ∗∗∗*p* < 0.001, ∗∗*p* < 0.01, ∗*p* < 0.05. *A*, Absolute Aβ, 2-WAY ANOVA, interaction F(8,30) = 26.14, *p* < 0.0001, row factor F(4,30) = 69.42, *p* < 0.0001, column factor F(2,30) = 44.53, *p* < 0.0001, (*C*) Absolute Aβ, 2-WAY ANOVA, interaction F(10,36) = 8.300, *p* < 0.0001, row factor F(5,36) = 16.76, *p* < 0.0001, column factor F(2,36) = 7.373, *p* < 0.001, (*E*) Absolute Aβ, 2-WAY ANOVA, interaction F(6,24) = 7.742, *p* < 0.001, row factor F(3,24) = 6.049, *p* < 0.01, column factor F(2,24) = 9.651, *p* < 0.001, (*G*) Absolute Aβ, 2-WAY ANOVA, interaction F(8,30) = 11.01, *p* < 0.0001, row factor F(4,30) = 13.70, *p* < 0.0001, column factor F(2,30) = 21.08, *p* < 0.0001.

In terms of Aβ species, the levels of Aβ40 and Aβ42 increased by ∼60% each when BACE1-SH-SY5Y cells were treated with 10^−8^ M VBA2092464 (R3) (Fig. 2, *F* and *G*). At 10^−7^ M VBA2092464 (R3), Aβ34 levels were notably and significantly decreasing (plateaued by 10^−6^ M), whereas Aβ40 and Aβ42 reached maximal levels (∼115% increase each; Fig. 2, *F* and *G*). For the BACE1 inhibitors with different pKa values (N6, N8, N10, and N11), the corresponding 10^−7^ M treatments yielded similar trends in the Aβ levels and ratios (*i.e.*, decreased Aβ34 vs. increased Aβ40 and Aβ42) as observed for VBA2092464 (R3) (compare Figs. 2, 3, and Supporting information S3). Unlike in WT-SH-SY5Y cells, BACE1 inhibitors consequently increased Aβ40/Aβ34 and Aβ42/Aβ34 ratios in BACE1-SH-SY5Y cells at high inhibitor concentrations (compare Fig. 1, *I* and *J* with Fig. 2, *I* and *J* and Supporting information S3). Taken together, our BACE1-SH-SY5Y cell results indicate that all inhibitor treatments (VBA2092464 (R3), N6, N8, N10, and N11) resulted in similarly altered Aβ metabolism. At the critical threshold (10^−7^ M inhibitor concentrations), Aβ clearance was decreased as indicated by reduced Aβ34 levels and elevated Aβ40 and Aβ42 levels.

### *In vitro* model #3—inhibition of BACE1 activity in stable APP-C99-overexpressing SH-SY5Y cells

**Figure 4:**
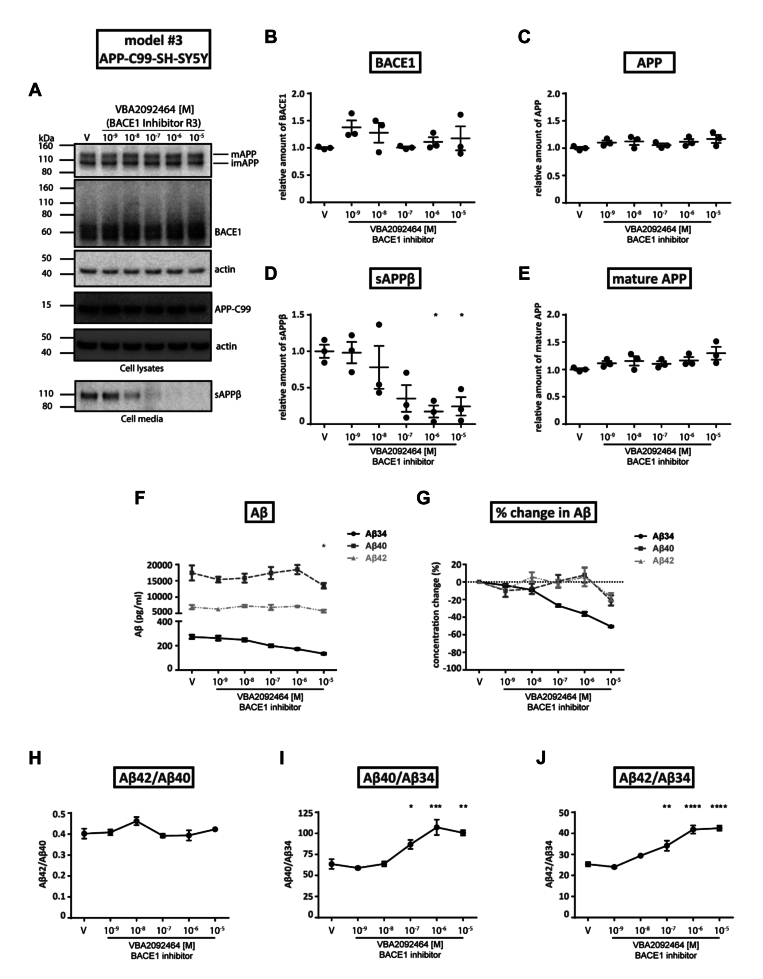
**Effect of BACE1 inhibitor VBA2092464 (R3) on *stably APP-C99 overexpressing SH-SY5Y cells.*** APP-C99-SH-SY5Y cells were treated with vehicle (V) or varying concentrations of the VBA2092464 (R3) BACE1 inhibitor. *A*, Western blotting (representative of three independent experiments) of cell lysates for the detection of APP (*upper band*, mature; *lower band*, immature), BACE1, APP-C99 and actin (control), or cell media for secreted sAPPβ. *B–E*, conversion of WB data to relative amounts of BACE1, APP, sAPPβ, or mature APP as a function of BACE1 inhibitor concentration. *F*, quantification of Aβ34, Aβ40, and Aβ42 levels in cell media as a function of BACE1 inhibitor concentration as determined by ELISA. *G*, conversion of ELISA data (pg/ml) to the percentage change of Aβ levels (relative to the vehicle condition). *H*–*J*, conversion of ELISA data (pg/ml) to specified Aβ ratios. Statistics: Bars and error bars indicate mean ± s.e.m. Dunnet’s *post hoc* tests were performed for pairwise comparisons; selected comparisons are highlighted ∗∗∗∗*p* < 0.0001, ∗∗∗*p* < 0.001, ∗∗*p* < 0.01, ∗*p* < 0.05. *B*, BACE1, 1-WAY ANOVA, F(5,12) = 1.280, *p* = 0.3344, (*C*) APP, 1-WAY ANOVA, F(5,12) = 1.419, *p* = 0.2860, (*D*) sAPPβ, 1-WAY ANOVA, F(5,12) = 4.904, *p* < 0.05, (*E*) mature APP, 1-WAY ANOVA, F(5,12) = 1.965, *p* = 0.1568, (*F*) Absolute Aβ, 2-WAY ANOVA, interaction F(10,36) = 1.006, *p* = 0.4573, row factor F(5,36) = 2.045, *p* = 0.0955, column factor F(2,36) = 451.5, *p* < 0.0001, (*H*) Aβ42/Aβ40, 1-WAY ANOVA, F(5,12) = 1.807, *p* = 0.1860, (*I*) Aβ40/Aβ34, 1-WAY ANOVA, F(5,12) = 15.79, *p* < 0.0001, (*J*) Aβ42/Aβ34, 1-WAY ANOVA, F(5,12) = 31.73, *p* < 0.0001.

**Figure 5:**
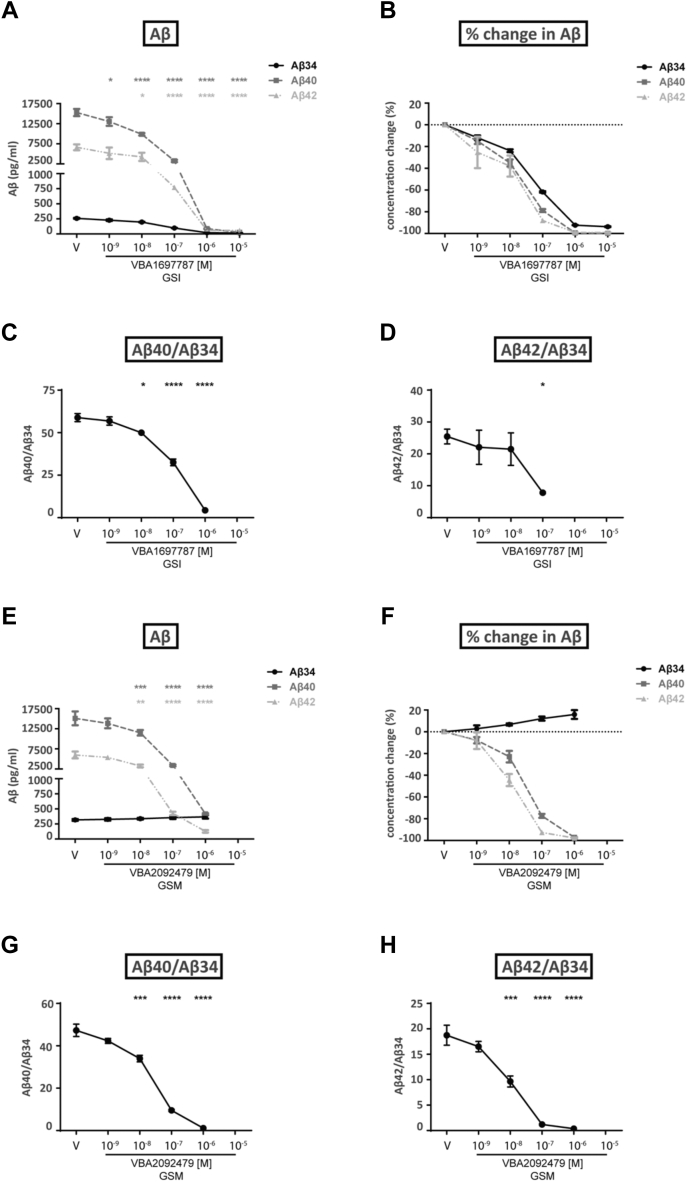
**Effects of γ-secretase inhibitor VBA1697787 (R1) and γ-secretase modulator VBA2092479 (R2) on *stably APP-C99 overexpressing SH-SY5Y cells*.** APP-C99-SH-SY5Y cells were treated with vehicle (V) or varying concentrations of the VBA1697787 (GSI R1) (*A*–*D*) or VBA2092479 (GSM R2) (*E*−*H*) compounds. Three independent experiments were performed. *A and E*, quantification of Aβ34, Aβ40 and Aβ42 levels in cell media as a function of γ-secretase inhibitor/modulator concentrations as determined by ELISA. b and f. Conversion of ELISA data (pg/ml) to the percentage change of Aβ levels (relative to the vehicle condition). *C, D, G*, and *H*. conversion of ELISA data (pg/ml) to specified Aβ ratios. Statistics: Bars and error bars indicate mean ± s.e.m. Dunnet’s *post hoc* tests were performed for pairwise comparisons; selected comparisons are highlighted ∗∗∗∗*p* < 0.0001, ∗∗∗*p* < 0.001, ∗∗*p* < 0.01, ∗*p* < 0.05. *A*, Absolute Aβ, 2-WAY ANOVA, interaction F(10,36) = 34.14, *p* < 0.0001, row factor F(5,36) = 96.84, *p* < 0.0001, column factor F(2,36) = 218.1, *p* < 0.0001, (*C*) Aβ40/Aβ34, 1-WAY ANOVA, F(4,10) = 167.1, *p* < 0.0001, (*D*) Aβ42/Aβ34, 1-WAY ANOVA, F(3,8) = 4.049, *p* = 0.05, (*E*) Absolute Aβ, 2-WAY ANOVA, interaction F(10,36) = 36.03, *p* < 0.0001, row factor F(5,36) = 90.07, *p* < 0.0001, column factor F(2,36) = 236.3, *p* < 0.0001, (*F*) Aβ40/Aβ34, 1-WAY ANOVA, F(4,10) = 164.8, *p* < 0.0001, (*G*) Aβ42/Aβ34, 1-WAY ANOVA, F(4,10) = 58.65, *p* < 0.0001.

To study the effect of inhibitors specifically on the amyloidolytic activity of BACE1, stable APP-C99-overexpressing SH-SY5Y (APP-C99-SH-SY5Y) cells were utilized. In this model, the amyloidogenic cleavage of BACE1 is bypassed as the product of this cleavage, APP-C99, is overexpressed. Therefore, the BACE1 inhibitor VBA2092464 (R3) was expected to act mainly on the amyloidolytic activity of BACE1. Since APP-C99 is a direct substrate of γ-secretase, we also tested the effects of a γ-secretase inhibitor (GSI; VBA1697787, termed R1) and a γ-secretase modulator (GSM; VBA2092479, termed R2). In the absence of any inhibitors, levels of Aβ42, Aβ40, and Aβ34 were higher (by ∼120-, ∼70-, and ∼20-fold respectively) in APP-C99-SH-SY5Y cells compared to wild-type SH-SY5Y cells (compare Figs. 1*F*, 4*F*, 5, *A*, and *E*). As observed for wild-type SH-SY5Y cells (Fig. 1*A*), the levels of BACE1 and full-length mature APP expression remained unaltered in APP-C99-SH-SY5Y cells when treated (10^−9^ M to 10^−5^ M) with the BACE1 inhibitor VBA2092464 (R3; Fig. 4, *A*–*E*), γ-secretase inhibitor VBA1697787 (GSI R1; Supporting information S4*A*), or γ-secretase modulator VBA2092479 (GSM R2; Supporting information S4*B*).

In terms of Aβ species, the BACE1 inhibitor VBA2092464 (R3) significantly reduced Aβ34 levels (∼40% maximum) in APP-C99-SH-SY5Y cells at concentrations of 10^−8^ M to 10^−6^ M (Fig. 4, *F*–*J*). Over the same concentration range, the very high Aβ40 and Aβ42 levels produced by the APP-C99-SH-SY5Y cells were unaltered yet reduced by ∼20% at the highest 10^−5^ M concentration of VBA2092464 (R3) tested (Fig. 4*G*). Aβ40/Aβ34 and Aβ42/Aβ34 ratios significantly increased with 10^−7^ M to 10^−5^ M treatment of VBA2092464 (R3) showing the decrease in Aβ34 levels, *i.e.*, inhibition of amyloidolytic activity of BACE1 (Fig. 4, *H*–*J*). In the same cells, over a non-toxic concentration range of 10^−9^ M to 10^−5^ M (Supporting information S1), the γ-secretase inhibitor VBA1697787 (GSI, R1) completely abolished Aβ34, Aβ40, and Aβ42 production by 10^−6^ M (Fig. 5, *A* and *B*). Over non-toxic 10^−9^ M to 10^−6^ M concentrations (Supporting information S1), the γ-secretase modulator VBA2092479 (GSM, R2) sharply decreased Aβ40 and Aβ42 levels by 10^−6^ M as previously reported by Satir and co-workers (20) but Aβ34 increased by 20% over the same concentration range (Fig. 5, *E*–*H*). Unlike treatment with VBA2092464 (R3), Aβ40/Aβ34 and Aβ42/Aβ34 ratios significantly decreased upon treatments with VBA1697787 (GSI, R1) starting at 10^-8^ M and VBA2092479 (GSM, R2) (10^−8^ M to 10^−6^ M) (Fig. 5, *C*, *D*, *G*, and *H*). Thus, BACE1 amyloidolytic activity was not impaired by VBA2092479 (GSM, R2) since Aβ34 was continuously produced by the γ-secretase in the presence of VBA2092479 (GSM, R2).

### *In vitro* model #4—amyloidolytic activity of recombinant BACE1 with synthetic Aβ40 and Aβ42 peptides

**Figure 6:**
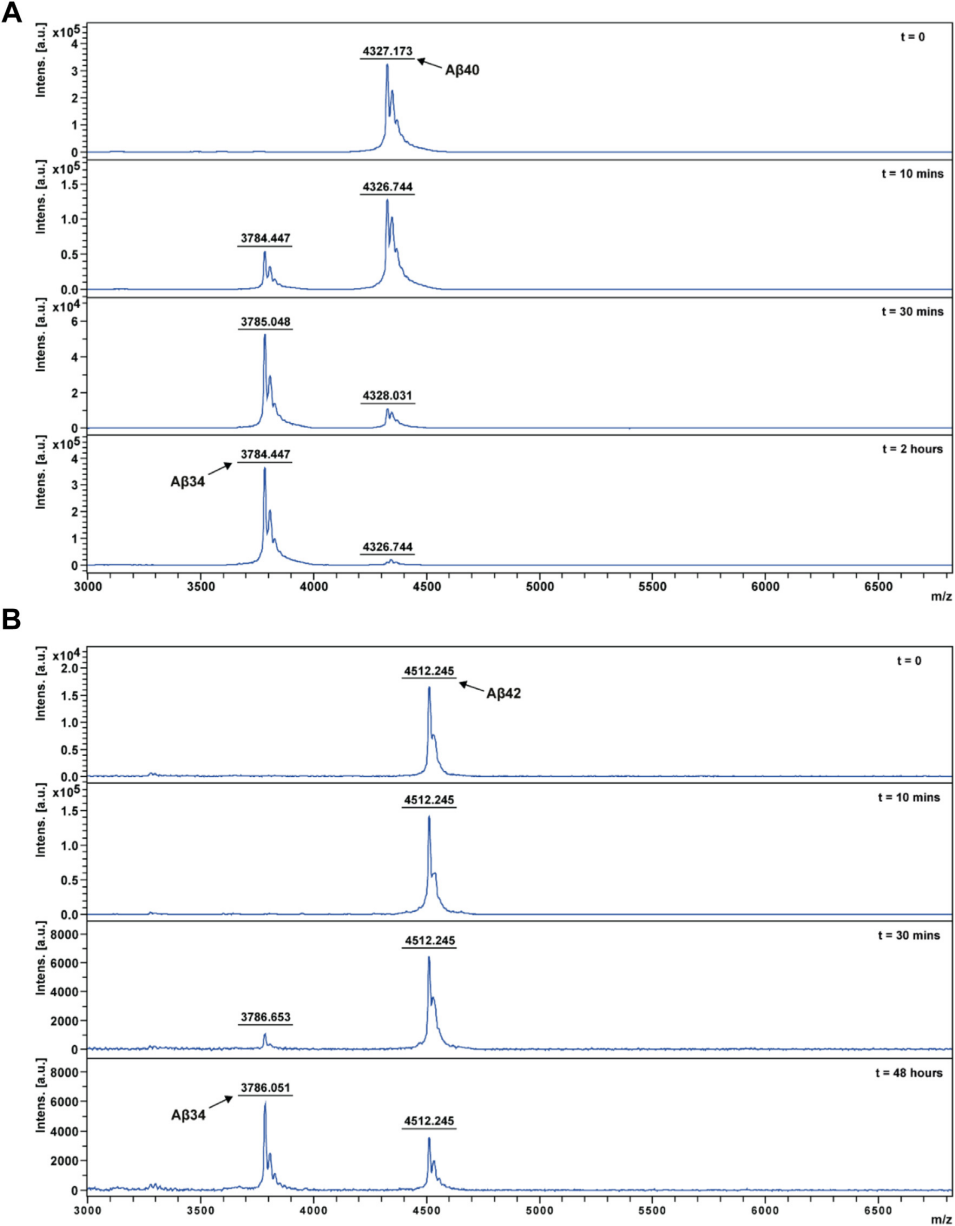
**MALDI-TOF analysis of BACE1-cleaved peptides**. *In vitro* digestion of (*A*) Aβ40 or (*B*) Aβ42 by BACE1. Synthetic peptides (50 μg/ml of Aβ40 (equals 11.6 μM) and Aβ42 (equals 11.1 μM)) were incubated with 10 μg/ml recombinant BACE1 (0.14 μM) at 37 °C for varying time points as indicated. *A*, representative MALDI-TOF spectra (*top to bottom*: time = 0, 10 min, 30 min, and 2 h) to monitor the cleavage of Aβ40 substrate (4327 *m/z*) to Aβ34 product (3785 *m/z*) by BACE1 enzyme. *B*, representative MALDI-TOF spectra (*top to bottom*: time = 0, 10 min, 30 min, and 48 h) to monitor the cleavage of Aβ42 substrate (4512 *m/z*) to Aβ34 product (3786 *m/z*) by BACE1 enzyme. Modest differences between the observed peaks and theoretical monoisotopic masses (Aβ34: 3784.9 + 1H+ = 3785.9 Da; Aβ40: 4327.1 + 1H+ = 4378.1 Da; Aβ42: 4511.3 + 1H+ = 4512.3 Da) were due to the linear detection method used to maximize MALDI-TOF assay sensitivity.

**Figure 7:**
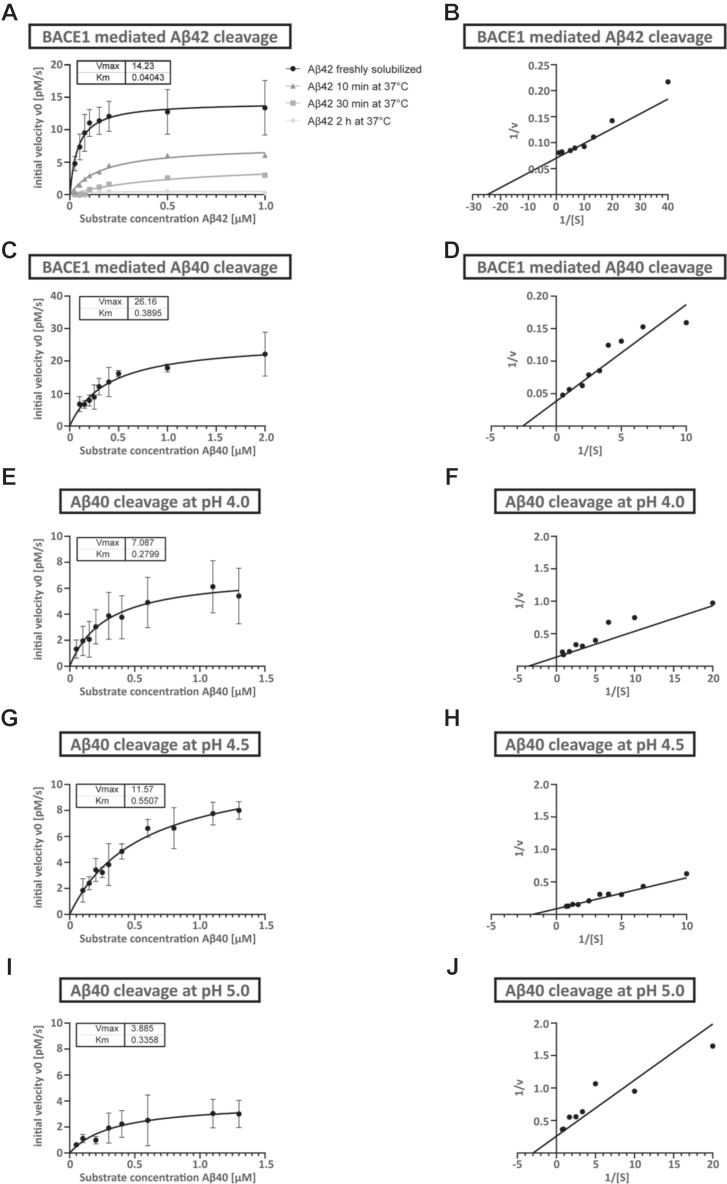
**Kinetics of BACE1 cleavage of Aβ40 and Aβ42 and the effect of pH.** Michaelis-Menten kinetics calculated for BACE1-mediated cleavage of freshly solubilized Aβ42 and kinetic curves of Aβ42 incubated for times as indicated (*A*), the Lineweaver-Burk plot for freshly solubilized Aβ42 (*B*), Michaelis-Menten kinetics for Aβ40 (*C*) at different pHs (*E*, *G* and *I*) with Vmax and Km values indicated on respective graphs. Lineweaver-Burk plots for Aβ40 are shown in (*D, F, H*, and *J*). Synthetic Aβ peptides were diluted to indicated concentrations and incubated with a constant concentration of BACE1. The initial reaction velocities (v0) were determined by measuring the product (Aβ34) concentrations over time by ELISA. Initial velocities were plotted against the used substrate concentrations and fitted using the Michaelis-Menten equation. Mean of three independent experiments is shown. Bars and error bars indicate mean ± SD.

To explain the biased degradation towards Aβ40 and the altered ratio in favor of increased Aβ42 (Fig. 1*H*), we incubated recombinant BACE1 enzyme with synthetic Aβ40 or Aβ42 peptide substrates and analyzed the cleavage products by MALDI-TOF mass spectrometry (Fig. 6). Whereas the majority of Aβ40 was converted to Aβ34 product after only 30 min (Fig. 6*A*), the majority of Aβ42 was converted to Aβ34 product only after longer incubation periods of 48 h (Fig. 6*B*). Complementary kinetic analyses using the same recombinant BACE1 and synthetic Aβ peptides revealed that the Michaelis constants (K_M_; concentration of substrate when the rate is half of the maximum velocity) for both substrates were in the nanomolar range: The K_M_ for Aβ42 was 40.4 nM and 389.5 nM for Aβ40 (Fig. 7, *A* and *C*). In line with its high affinity towards Aβ42, BACE1 showed a higher catalytic efficiency (k_cat_/K_M_) towards monomerized and freshly dissolved Aβ42 than Aβ40, with efficiencies of 5028.1 M^−1^s^−1^ and 959.7 M^−1^s^−1^ (calculated from Fig. 7, *A* and *C*), respectively. Furthermore, incubating Aβ42 at 37 °C before the kinetic assay and at time points as indicated demonstrated that its degradation is attenuated highly likely due to oligomerization of Aβ42 (Fig. 7*A*). In agreement with concentrations of the peptides used, Aβ42 has been described to form oligomers within minutes when the monomer concentration is in the micromolar (μM) range (33). The K_M_ was not determined for incubated Aβ42 since it would not be possible to assign measured values to specific oligomeric states.

Comparing the catalytic efficiency at different pHs with Aβ40 as a substrate, the maximum velocity (V_max_) of Aβ40 cleavage by BACE1 was highest at pH 4.5 (11.6 pM/s) (Fig. 7, *E*, *G* and *I*). Notably, the catalytic efficiency was highest at pH 4.0 (361.7 M^−1^s^−1^) as compared to pH 4.5 (300.1 M^−1^s^−1^) and pH 5.0 (165.3 M^−1^s^−1^) (Fig. 7, *E*, *G*, and *I*). Lineweaver-Burk plots for kinetics are shown in Figure 7, *B*, *D*, *F*, *H*, and *J*.

Overall, our kinetic analyses demonstrate that (i) the affinity of BACE1 for Aβ substrates (K_M_ = nanomolar range) is greater than for APP (K_M_ = micromolar range; (34, 35), (ii) BACE1 has a higher affinity and catalytic efficiency for Aβ42 than Aβ40, and (iii) the aggregation of Aβ42 decreases its cleavage efficiency by BACE1.

## Discussion

Since the discovery that BACE1-mediated cleavage of APP is the rate-limiting step for Aβ production, BACE1 has remained a well-validated therapeutic target for AD (36). While the field has traditionally focused on the development of BACE1 inhibitors to attenuate the “amyloidogenic” conversion of APP to clinically relevant Aβ40 (vascular amyloid) and Aβ42 (neuritic plaques), clinical trials have not improved cognition and/or were halted due to adverse off-target effects or safety concerns. As our fundamental understanding of BACE1 biology continues to rapidly evolve, including the effect of inhibitors on other BACE1 substrates essential for axon guidance and synaptogenesis (37), more selective BACE1 inhibitors are needed to avoid the adverse side effects encountered to date.

**Figure 8:**
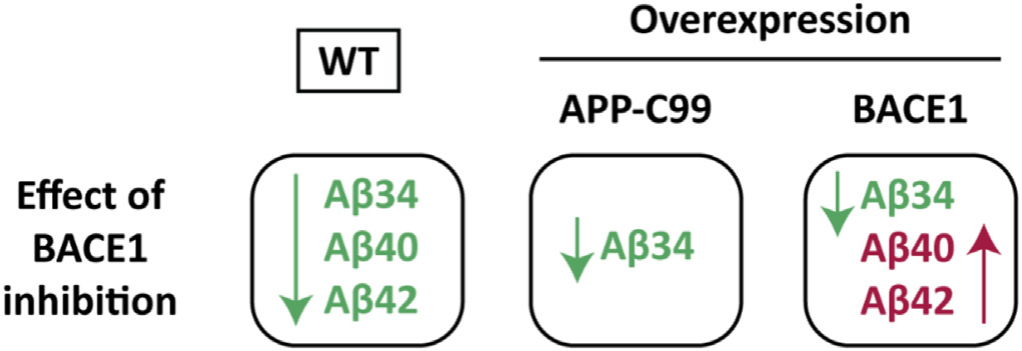
**Schematic overview of effects of BACE1 inhibition in three cellular models tested.** Inhibition of BACE1 affected Aβ42, Aβ40 and Aβ34 levels depending on the substrate to enzyme (Aβ:BACE1) ratio. In WT cells (model#1) with endogenous levels of substrate and enzyme, BACE1 inhibition decreased levels of all three Aβ peptides tested. At high levels of substrates, *i.e.*, Aβ42 and Aβ40 (model#3: APP-C99 overexpression bypasses the amyloidogenic activity of BACE1), Aβ34 levels are reduced while Aβ42 and Aβ40 levels remain unaffected. BACE1 inhibition under BACE1 overexpression condition (model#2) results in reduced Aβ34 levels and a significant concomitant increase of Aβ40 and Aβ42 levels indicating that amyloidolytic activity of BACE1 is more susceptible to inhibition over a certain concentration range than its amyloidogenic activity.

To better predict the mechanism of action for candidate AD therapeutics, the current study was specifically designed to test the effect of BACE1 inhibitors, γ-secretase inhibitors (GSI), and γ-secretase modulators (GSM) on amyloid homeostasis. Our head-to-head comparison tested the secretase-targeting compounds on APP processing (sAPPβ), Aβ production (Aβ40, Aβ42), and Aβ clearance (Aβ34) in human neuroblastoma SH-SY5Y cells under varying BACE1/APP expression levels. We summarized our main findings in Figure 8. Understanding how candidate inhibitors affect *both* the amyloidogenic (Aβ production) and amyloidolytic (Aβ clearance) activities of BACE1 may explain why BACE1 inhibitors have yielded variable “high-dose” (>50% Aβ inhibition) vs. “low-dose” (<50% Aβ inhibition) outcomes.

Under endogenous expression levels of BACE1 and APP (*i.e.*, wild-type SH-SY5Y cells as model #1), the BACE1-selective VBA2092464 (R3) compound inhibited the amyloidogenic activity of BACE1, as evidenced by the significant decrease in sAPPβ levels by 10^-7^ M VBA2092464 (R3). At the matching inhibitor concentration, the decreased Aβ34 levels and increasing Aβ42/Aβ40 ratio indicated that the amyloidolytic activity of BACE1 was impaired (*i.e.*, reduced Aβ clearance). To explain the biased degradation of Aβ40 and altered ratio in favor of increased Aβ42, our cleavage analysis (MALDI-TOF data) showed that synthetic Aβ40 was degraded to Aβ34 by BACE1 more quickly compared to oligomeric Aβ42. Complementary kinetic (Michaelis-Menten) analyses showed that although BACE1 has a higher affinity and catalytic efficiency towards monomerized Aβ42, the cleavage kinetics of Aβ42 are strongly attenuated by the incubation time and the accompanying oligomerization. In wild-type SH-SY5Y cells, the immediate decrease of Aβ40 levels increased the Aβ42/Aβ40 ratio from 0.2 to 0.3. Consistent with our findings, a clinical trial with the BACE1 inhibitor CNP520 found that the Aβ42/Aβ40 ratio increased significantly at higher doses while no change was observed in the placebo and lower dose groups (38). However, *in vivo*, BACE1 inhibitors that increase the Aβ42/Aβ40 ratio could be problematic, especially at preclinical stages (39).

In stably BACE1-overexpressing SH-SY5Y cells (model #2), elevated levels of all three Aβ species were detected in the presence of low nM concentrations of VBA2092464 (R3) (<10^−7^ M). At 10^−7^ M, however, Aβ42 and Aβ40 levels were significantly increased and Aβ34 was significantly decreased through inhibition of BACE1 amyloidolytic activity. Significantly decreased sAPPβ levels (when BACE1 amyloidogenic activity was inhibited) showed that elevated levels of Aβ42 and Aβ40 in the presence of 10^−7^ M VBA2092464 (R3) are not the result of the increased amyloidogenic activity (*i.e.*, increased production of Aβ42 and Aβ40) but the result of inhibited amyloidolytic activity. Intermittently high levels of such aggregation-prone species may represent a critical tipping point (progression from oligomers to plaques) in AD pathogenesis. All other BACE1 inhibitors tested in this study (*i.e.*, N6, N8, N10, and N11) yielded similar outcomes to VBA2092464 (R3) at the critical 10^-7^ M threshold.

In stably APP-C99 overexpressing SH-SY5Y cells (model #3: by-pass initial BACE1-mediated β-site cleavage of APP to focus on amyloidolytic activity), there were two notable differences in BACE1 inhibition compared to models #1 and #2 (as detailed above): (i) Aβ34 levels continued to steadily decrease at VBA2092464 (R3) concentrations above 10^−7^ M, and (ii) Aβ42 and Aβ40 levels were modestly decreased at only the highest VBA2092464 (R3) concentration (10^−5^ M) tested. In contrast, the γ-secretase inhibitor (GSI) inhibited the production of all three Aβ species as evidenced by their significantly reduced levels at the 10^−7^ M VBA1697787 (GSI, R1) threshold. While the γ-secretase modulator (GSM) abolished the production of Aβ42 and Aβ40 at 10^−7^ M VBA2092479 (GSM, R2), the level of Aβ34 was slightly increased under similar conditions. At endogenous levels of BACE1 in stably APP-C99 overexpressing cells treated with VBA2092464 (R3), the Aβ42/Aβ40 ratio similarly varied between 0.4 and 0.5 (Fig. 4*H*) as found in human CSF (32).

Overall, our findings indicate that both the amyloidogenic and amyloidolytic activities of BACE1 must be carefully considered when evaluating new candidate modulators. For example, existing BACE1 inhibitors were shown to exert distinct effects on neuronal Aβ metabolism and yielded a unique pattern for each secreted Aβ peptide in CSF from dogs by immunoprecipitation-mass spectrometry (40) including an unexpected decrease in Aβ34 levels at that time. Our current results provide a concrete explanation for that outcome, *i.e.*, BACE1 inhibitor inadvertently affected BACE1 amyloidolytic activity. The BACE1 inhibitor effect is unique as such findings are in sharp contrast to γ-secretase modulators.

Our complementary SH-SY5Y models demonstrate that testing varying BACE1/APP expression levels is critical to better understanding the *in vivo* effects of BACE1 inhibitors on Aβ homeostasis. Higher protein levels of BACE1 have been reported in the normal aging brain and to an even larger extent in the AD brain (27, 41, 42). BACE1 activity in serum was found ∼30% higher in patients with AD and vascular dementia (VD) compared to controls (26, 43, 44). These studies suggest that BACE1 is highly active before the onset of overt dementia, and BACE1 amyloidolytic activity might be a defense mechanism to decrease the level of aggregation-prone Aβ40 and Aβ42. Therefore, any moderate slowdown of amyloid clearance by BACE1 inhibitors (“high-dose” or “low-dose” strategies) may negatively impact clinical outcomes by promoting the increased accumulation of aggregation-prone Aβ42 and Aβ40.

## Experimental procedures

### Secretase-targeting compounds

BACE1 inhibitors were from Roche (VBA2092464; R3), and Novartis (N6, N8, N10 and N11). Both the γ-secretase inhibitor (VBA1697787; GSI R1) and γ-secretase modulator (VBA2092479; GSM R2) were from Roche. All compounds were prepared as 100 mM stocks in 100% DMSO.

The toxicity of all secretase-targeting compounds was assessed (MTT assay using wild-type SH-SY5Y cells) before performing any of the reported experiments at non-toxic concentrations only. As noted in Supporting information S1, compounds R1, R3, and N8 were non-toxic overall concentrations tested, whereas others were toxic at 10^−5^ M (R2, N6, and N11) or 10^−6^ M (N10).

### Cell culture and compound treatments

A human BACE1 construct (full-length BACE1, isoform A; pcDNA3.1+/Zeo; Invitrogen), APP695 (with an N-terminal Myc-tag; pcDNA3.1+/Zeo; Invitrogen) or APP-C99 construct (with a C-terminal FLAG-tag; pcDNA3.1+/Zeo; Invitrogen) were used to generate human neuroblastoma (SH-SY5Y) cells that stably overexpress BACE1 or APP-C99.

Wild-type human neuroblastoma (SH-SY5Y) cells (DSMZ No. ACC209; DSMZ) or stably overexpressing BACE1 or APP-C99 cells were cultured in DMEM/F12 (10% fetal bovine serum (FBS), 2 mM L-glutamine, 1 mM sodium pyruvate) in a humidified incubator at 37 °C, 5% CO_2_. Hygromycin B (250 μg/ml; Millipore) was used to select stable cell lines. Cells were seeded at 300,000 cells/well (6-well plates; Fisher Scientific) and then treated with vehicle (control) or increasing compound concentrations (BACE1 inhibitor, GSI, or GSM) after 24 h. The cells were harvested at 72 h post-treatment.

### Thiaxolyl blue tetrazolium bromide (MTT) cytotoxicity assay

Wild-type SH-SY5Y cells were seeded in 96-well plates (Fisher Scientific) and cultured for 24 h before treating with vehicle (control) or increasing compound concentrations for 72 h. MTT was prepared at 5 mg/ml in PBS, filtered, and then incubated at 37 °C for 15 min prior to all assays. MTT was added to the treated wells at a 1:10 ratio for 1 h at 37 °C until the blue color was observed under the microscope. The media was aspirated and 100 μl DMSO was added to the wells. The plates were shaken for 5 min. Using Synergy H1, BioTek Instruments Inc plate reader, absorbance at 562 nm was recorded.

### Sample preparation

For all experiments performed, cells were harvested on ice. Conditioned media were centrifuged at 425 g at 4 °C for 10 min. Levels of Aβ34, Aβ40, and Aβ42 in conditioned media were quantified by ELISA or MSD. sAPPβ levels in cell media were detected by Western blot. After cell media were collected, cells were washed with cold PBS before incubated and lysed with Whole Cell Extract Buffer (25 mM HEPES (pH 7.7), 0.3 M NaCl, 1.5 mM MgCl_2_, 0.2 mM ethylenediaminetetraacetic acid, 0.1% Triton-X-100, 0.5 mM dithiothreitol, 4 mM NaF, 0.1 mM Na_3_VO_4_, 1 mM PMSF, Complete Protease Inhibitor Cocktail (Roche)) at 4 °C for 60 min. Nuclear material was cleared from the cell lysates by centrifugation at 10,600*g* at 4 °C for 15 min and protein levels were detected by Western blotting.

### Western blotting

Samples were prepared by adding LDS loading buffer and 2-mercaptoethanol to the cell lysates according to the protocol provided by the manufacturer (Invitrogen). The proteins were solubilized and denatured by heating the samples to 70 °C for 10 min 4 to 12% Bis-Tris gradient gels (Invitrogen) were used to separate the proteins, which were then transferred to 0.45 μm nitrocellulose (Biorad) or 0.45 μm polyvinylidene difluoride (PVDF) (Millipore) membranes at 400 mA at 4 °C for 2.5 h. Proteins were detected by the antibodies as indicated in the section below. The primary and secondary antibodies were used in either phosphate- or tris-buffered saline. Signals were recorded on ImageQuant LAS 500 and LAS 600 (GE Healthcare Life Sciences). Western blots were quantified on ImageJ and all protein levels were normalized to actin levels.

The primary antibodies used for Western blot analysis were the following: anti-BACE1 1:2000 dilution (monoclonal D10E5, Cell Signaling, Catalog #mAb5606), anti-actin 1:5000 dilution (Millipore, Catalog #MAB1501), anti-sAPPβ 1:2000 dilution (IBL, Catalog #JP18957), anti-APP ectodomain 22C11 1:10,000 dilution (Millipore, Catalog #MAB348), and anti-FLAG 1:1000 dilution (M2, Sigma-Aldrich, Catalog #F1804). The secondary antibodies used for Western blot analysis were the following: anti-mouse- and anti-rabbit-horseradish peroxidase 1:10,000 dilution (Promega, Catalog #W4021 (anti-mouse) and # W4011 (anti-rabbit)).

### Meso scale discovery assay

Custom-printed 4-plex plates were used as described previously (10). Briefly, 150 μl 5% meso scale discovery (MSD) Blocker A solution was used to block the plates for 1 h at room temperature with gentle shaking and plates were washed three times with 250 μl PBS-T (0.05% Tween). Peptide calibrators were diluted in MSD Diluent 35. Samples and calibrators were loaded together with SULFO-TAG 4G8 or 6E10 detection antibody diluted in MSD Diluent 100 to the plates. Plates were incubated overnight at 4 °C with gentle shaking. After three washes with 250 μl PBS-T, 150 μl MSD read buffer was added to the wells. Plates were read by an MSD QuickPlex SQ 120 Imager and data were analyzed by MSD Workbench software.

### Sandwich-based ELISA assay

96-well Nunc plates (Thermo Fisher Scientific Inc.) were coated with 5 μg/ml neo-epitope monoclonal anti-Aβ34 (in-house, 226 (10)), anti-Aβ40 (MABN11-KC Sigma-Aldrich, G2-10) or anti-Aβ42 (MABN13 Sigma-Aldrich, G2-13) capture antibody in 100 mM sodium carbonate (pH 9.6) and were incubated overnight at 4 °C with gentle shaking. Plates were washed 5 times for 10 min with PBS-T washing buffer (1.1 mM NaH_2_PO_4_, 8.5 mM Na_2_HPO_4_, 13.7 mM NaCl, (pH 7.4), 0.1% Tween-20). For blocking, plates were incubated with 250 μl Stabil CoatImmunoassay Stabilizer (SurModics Inc) for 2 h at room temperature with gentle shaking. 50 μl sample (cell media) or calibrator (synthetic peptide standards diluted in DMEM or DMEM/F12) were loaded to the wells together with 50 μl of 0.075 μg/ml detection antibody (W02-biotin) in assay buffer (90% 11 mM NaH_2_PO_4_, 85 mM Na_2_HPO_4_, 137 mM NaCl, (pH 7.4), 0.5% Tween-20, 1.5% BSA, 0.01% Thimerosal, and 10% SeaBlock blocking buffer (Thermo Fisher Scientific Inc.)) and plates were incubated overnight at 4°C with gentle shaking. Wells were washed 5 times for 10 min with PBS-T washing buffer. 100 μl Poly-HRP-conjugated-streptavidin (Pierce) (1:20,000 dilution) in Poly-HRP buffer (1.1 mM NaH_2_PO_4_, 8.5 mM Na_2_HPO_4_, 13.7 mM NaCl, (pH 7.4) 0.1% Tween-20, 5% BSA)) was added to the wells. Plates were incubated for 1 hour at room temperature with gentle shaking and washed 5 times for 10 min with PBS-T washing buffer. The enzymatic reaction was initiated by the addition of 100 μl 1-Step Ultra TMB-ELISA Substrate (Thermo Fisher Scientific Inc.) solution and the plates were incubated at room temperature in the dark for up to 30 min. To stop the reaction, 50 μl 1 M H_2_SO_4_, per well was added. Using Synergy H1, BioTek Instruments Inc. plate reader, absorbance at 450 nm and 630 nm (as a reference) was measured. The data analysis was performed with Gen5 BioTeksoftware. For the fitting of standard curves obtained from the absorbance of calibrators, the following a non-linear four-parameter logistic fit without weighting was used:

$$y=b_{2}+\frac{b_{1}-b_{2}}{1+{\left(\frac{x}{b_{3}}\right)}^{b_{4}}}$$

where y is signal, x is concentration, b_2_ is the estimated response at the infinite concentration, b_1_ is the estimated response at zero concentration, b_3_ is mid-range concentration and b_4_ is slope factor.

### Preparation of Aβ peptides

To monomerize the lyophilized Aβ42 and Aβ40 (Bachem Americas, Inc, 4014447, 4014442) concentrated formic acid was added (1:1 v/w). Aliquots of 20 μg were prepared. The formic acid was evaporated at 45 °C for 2 h in a SpeedVac Concentrator (SPD131DDA). To resolubilize the monomerized peptide, 1% ammonia water was added (1:2 v/w) after which the peptide was sonicated for 10 min (37 kHz, 4 °C). ddH20 was added (1:1 v/v) and the solution was sonicated for 10 min at the same settings. Peptide concentrations were confirmed using a NanoDrop OneC.

### *In vitro* cleavage

Freshly monomerized Aβ40 or Aβ42 peptides were diluted to 50 μg/ml in digestion buffer 1 (20 mM NaOAc, pH 4.5) and then incubated with 10 μg/ml soluble BACE1-Fc (ACRO Biosystems) at 37 °C. At timed intervals (minutes to hours), reaction volumes were sampled (10 μl each and kept on ice to avoid aggregation). To minimize Aβ peptide absorbance into the reaction tubes Eppendorf Protein LoBind tubes were used. BACE1-mediated cleavage of the Aβ40 or Aβ42 substrates to Aβ34 product was monitored by matrix-assisted laser desorption/ionization time-of-flight (MALDI-TOF) mass spectrometry. Briefly, time course samples were mixed 1:1 with HCCA matrix (Sigma #70990; prepared at 20 mg/ml in 50/50 solution of acetonitrile and 0.1% (v/v) trifluoroacetic acid) for spotting on polished steel targets. The co-crystallized samples were analyzed using an UltrafleXtreme MALDI-TOF/TOF system (Bruker Daltonics, Germany) in linear positive mode using FlexControl (v3.4 software). Ion intensities for sample sets were evaluated in FlexAnalysis (v3.4 software) by averaging three measurements of 4000 shots each (*i.e.* 12,000 shots total per sample).

### *In vitro* kinetics (Michaelis-Menten)

For the kinetic analyses of the BACE1 mediated Aβ40-and Aβ42 cleavage at pH 4.5, the resolubilized peptides were diluted to the desired peptide concentration (Aβ40: 0.1–2 μM, Aβ42: 0.025–1 μM) in digestion buffer 1. For the kinetic analyses with varying pH conditions, Aβ40 was diluted to the desired concentrations (0.05–1.5 μM) in digestion buffer 2 (100 mM citric acid, 200 mM Na_2_HPO_4_, pHs: 4.0, 4.5, 5.0, respectively). Truncated BACE1 (ACROBiosystems, BA1-H526) was dissolved in 0.01% (w/v) BSA to a concentration of 1 mg/ml, and 70 nM BACE1 was added to the peptide solutions.

All reaction mixtures were incubated at 37 °C and timed aliquots (between 2 and 45 min) were collected, diluted 1:10 in ice-cold StabilCoat, and kept on ice. Plots of increasing Aβ34 concentration (determined by ELISA) *versus* time were subjected to linear regression to determine the initial reaction velocities (v0). Subsequent plots of v0 *versus* substrate concentration were subjected to non-linear regression (fit to the Michaelis-Menten equation) to determine the maximum reaction velocities (V_max_) and Michaelis-Menten constants (K_M_). Catalytic activity (k_cat_) values were calculated as the quotient of maximum velocity and enzyme concentration.

### Statistical analyses

For all experiments, different conditions were analyzed by one-factor ANOVA (between-subject design) or two-factor ANOVA. Dunnet’s *post hoc* tests were performed for pairwise comparisons. GraphPad Prism 5 was used to run the statistical analysis.

## Data availability

All data are contained within the manuscript.

## Supporting information

This article contains supporting information.

## Conflict of interest

The authors declare that they have no conflicts of interest with the contents of this article.
