# Supplementary material for: Inhibition of BACE1 affected both its Aβ producing and degrading activities and increased Aβ42 and Aβ40 levels at high-level BACE1 expression
Source: J Biol Chem. 2024 Jun 27;300(8):107510. doi: 10.1016/j.jbc.2024.107510 (PMC11324814; doi:10.1016/j.jbc.2024.107510)
Supplement: Supporting Information 1.1 [file mmc1.pdf]

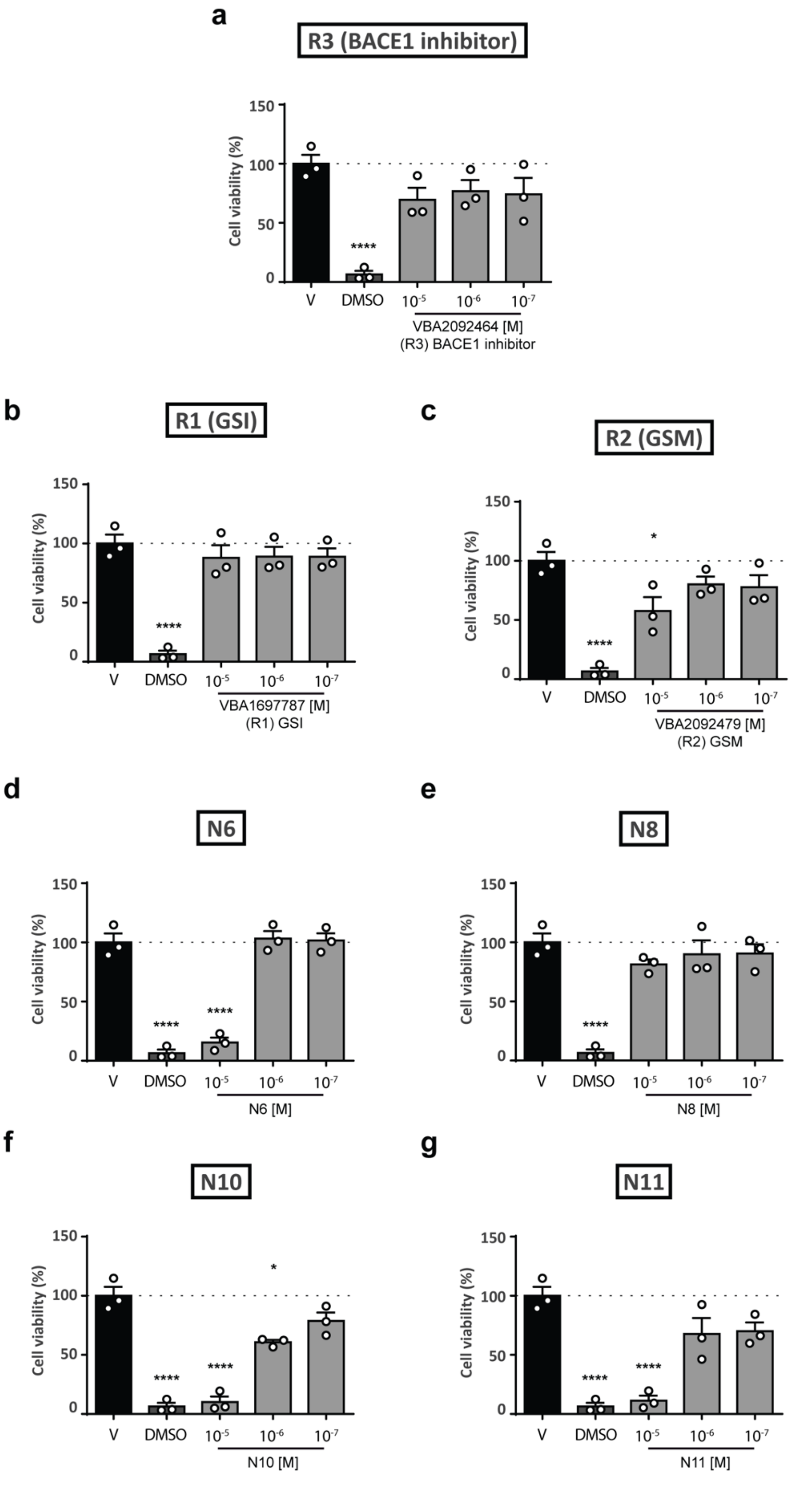

Supporting Information 1. MTT assay for all compounds in <u>WT SH-SY5Y cells</u>. WT SH-SY5Y cells were treated with indicated concentrations of the compounds. i.e., VBA2092464 (R3) BACE1 inhibitor (a), VBA1697787 (R1) GSI (b), VBA2092479 (R2) GSM (c), N6 (d), N8 (e), N10 (f) and N11 (g), to assess

toxicity. The data were collected from 3 independent experiments. For clarity, toxicity of each compound is depicted individually.

Statistics. Bars and error bars indicate mean ± s.e.m. Dunnet's post-hoc tests were performed for pairwise comparisons; selected comparisons are highlighted ****$p < 0.0001$, ***$p < 0.001$, **$p < 0.01$, *$p < 0.05$. 1-WAY ANOVA, $F(22,46) = 12.68$, $p < 0.0001$.
