# Supplementary material for: Inhibition of BACE1 affected both its Aβ producing and degrading activities and increased Aβ42 and Aβ40 levels at high-level BACE1 expression
Source: J Biol Chem. 2024 Jun 27;300(8):107510. doi: 10.1016/j.jbc.2024.107510 (PMC11324814; doi:10.1016/j.jbc.2024.107510)
Supplement: Supporting Information 2.2 [file mmc2.pdf]

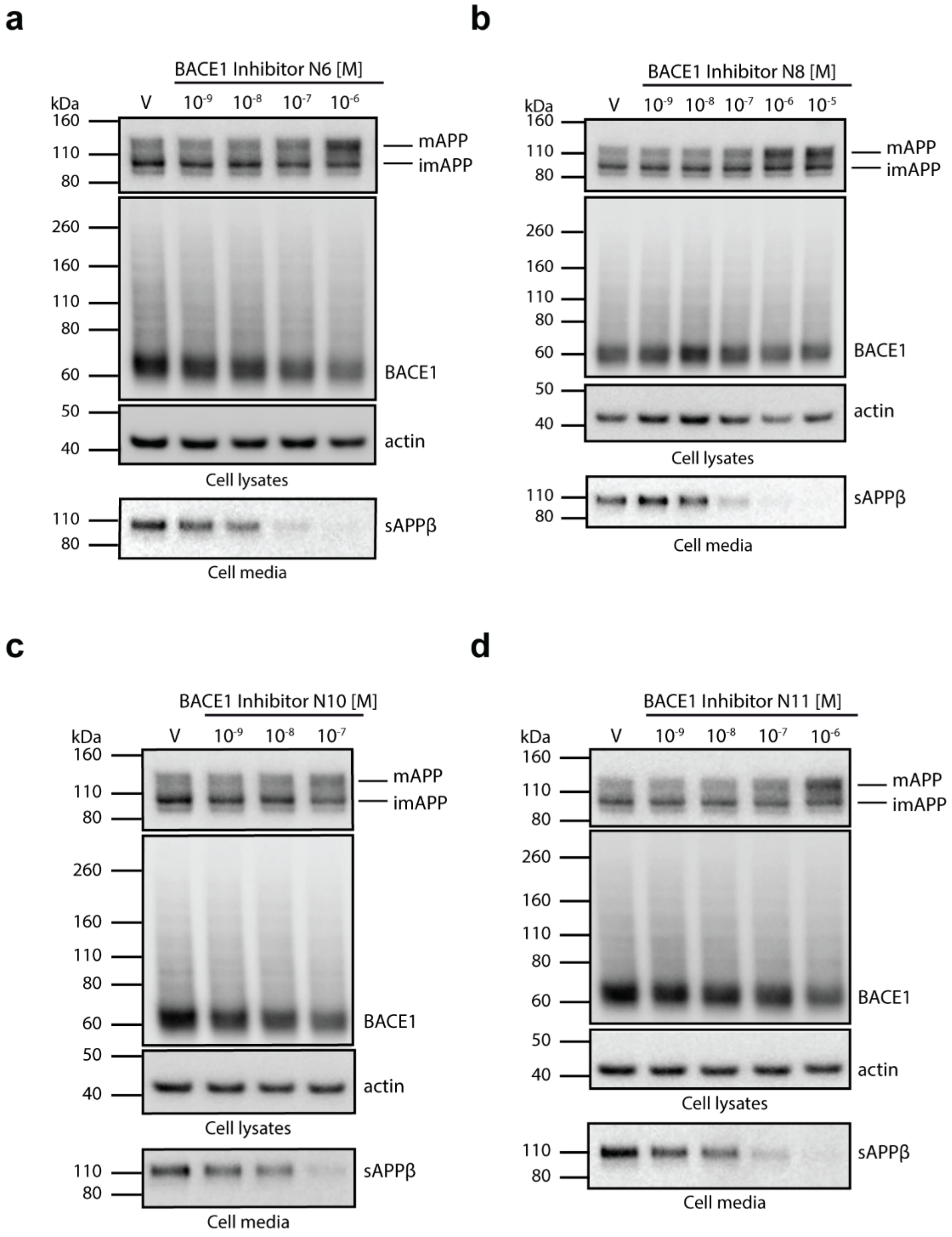

Supporting Information 2. Effect of BACE1 inhibitors N6, N8, N10 and and N11 on protein levels in stably BACE1 overexpressing SH-SY5Y cells. BACE1-SH-SY5Y cells were treated with vehicle (V) or varying concentrations of the N6 (a), N8 (b), N10 (c) or N11 (d) BACE1 inhibitor.

a – d. Western blotting (representative of 3 independent experiments) of cell lysates for the detection of APP (*upper band*, mature; *lower band*, immature), BACE1, and actin (control), or cell media for secreted sAPPβ.
