# Supplementary material for: Inhibition of BACE1 affected both its Aβ producing and degrading activities and increased Aβ42 and Aβ40 levels at high-level BACE1 expression
Source: J Biol Chem. 2024 Jun 27;300(8):107510. doi: 10.1016/j.jbc.2024.107510 (PMC11324814; doi:10.1016/j.jbc.2024.107510)
Supplement: Supporting Information 3.3 [file mmc3.pdf]

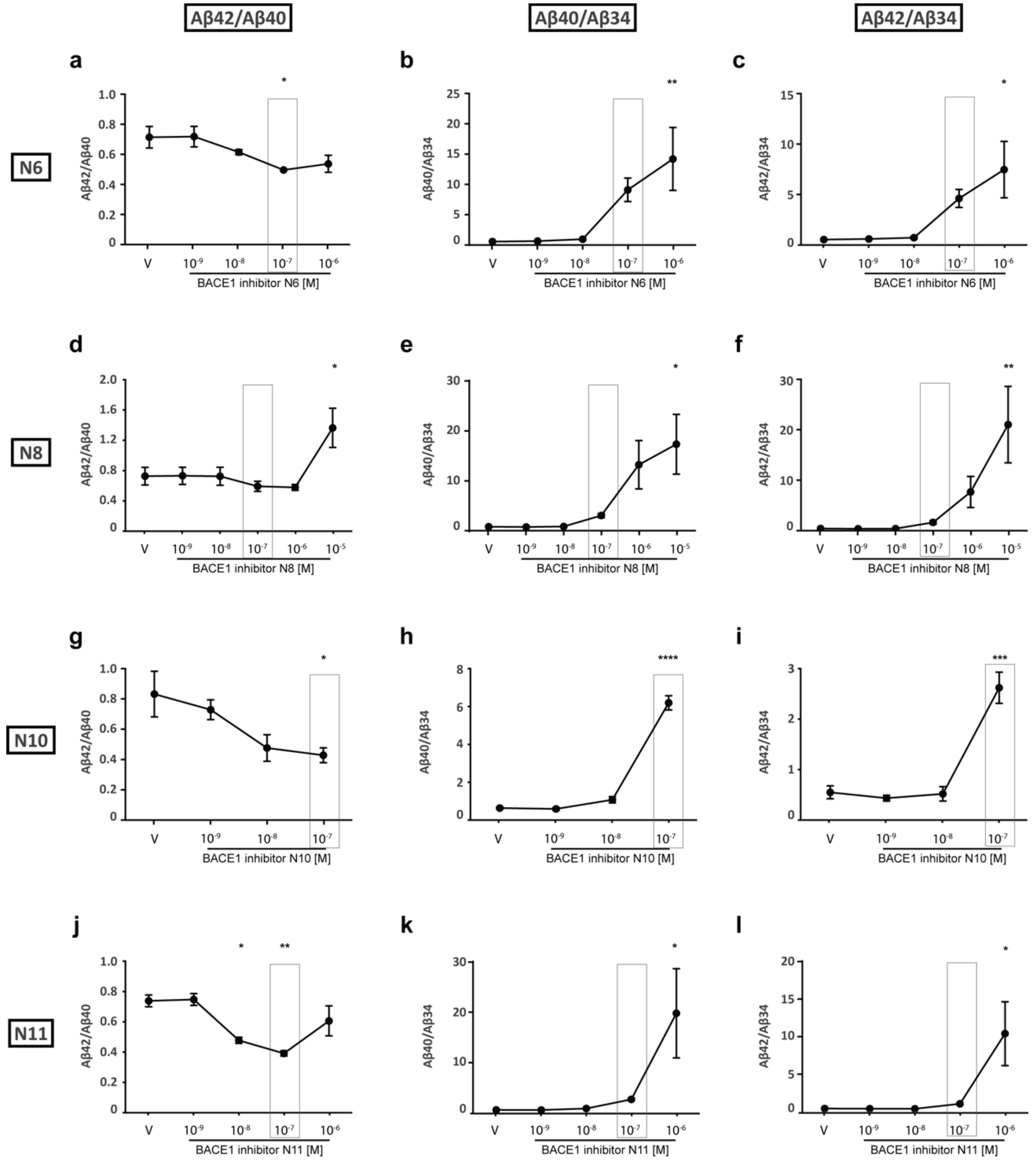

Supporting Information 3. Effects of BACE1 inhibitors that have different pKa values on Aβ ratios in <u>stably BACE1 overexpressing SH-SY5Y cells</u>. BACE1-SH-SY5Y cells were treated with vehicle (V) or varying concentrations of the N6, N8, N10 or N11 BACE1 inhibitor. 3 independent experiments were performed. Due to different toxicities of inhibitors, the concentrations covered are different. 10^{-7} M for all inhibitors is marked with a box in the figure.

a – l. Conversion of data absolute amounts (pg/mL) in Fig. 4 to specified Aβ ratios.

Statistics. Bars and error bars indicate mean ± s.e.m. Dunnet's post-hoc tests were performed for pairwise comparisons; selected comparisons are highlighted ****$p < 0.0001$, ***$p < 0.001$, **$p < 0.01$, *$p < 0.05$. (a)

Aβ42/Aβ40, 1-WAY ANOVA, $F(4,10) = 3.794$, $p < 0.5$, (b) Aβ40/Aβ34, 1-WAY ANOVA, $F(4,10) = 6.374$, $p < 0.01$, (c) Aβ42/Aβ34, 1-WAY ANOVA, $F(4,10) = 5.712$, $p < 0.05$, (d) Aβ42/Aβ40, 1-WAY ANOVA, $F(5,12) = 4.494$, $p < 0.05$, (e) Aβ40/Aβ34, 1-WAY ANOVA, $F(5,12) = 5.460$, $p < 0.01$, (f) Aβ42/Aβ34, 1-WAY ANOVA, $F(5,12) = 6.084$, $p < 0.01$, (g) Aβ42/Aβ40, 1-WAY ANOVA, $F(3,8) = 33.26$, $p < 0.0001$, (h) Aβ40/Aβ34, 1-WAY ANOVA, $F(3,8) = 164.9$, $p < 0.0001$, (i) Aβ42/Aβ34, 1-WAY ANOVA, $F(3,8) = 33.26$, $p < 0.0001$, (j) Aβ42/Aβ40, 1-WAY ANOVA, $F(4,10) = 9.074$, $p < 0.01$, (k) Aβ40/Aβ34, 1-WAY ANOVA, $F(4,10) = 4.448$, $p < 0.05$, (l) Aβ42/Aβ34, 1-WAY ANOVA, $F(4,10) = 5.371$, $p < 0.05$.
