# Supplementary material for: Inhibition of BACE1 affected both its Aβ producing and degrading activities and increased Aβ42 and Aβ40 levels at high-level BACE1 expression
Source: J Biol Chem. 2024 Jun 27;300(8):107510. doi: 10.1016/j.jbc.2024.107510 (PMC11324814; doi:10.1016/j.jbc.2024.107510)
Supplement: Supporting Information 4.4 [file mmc4.pdf]

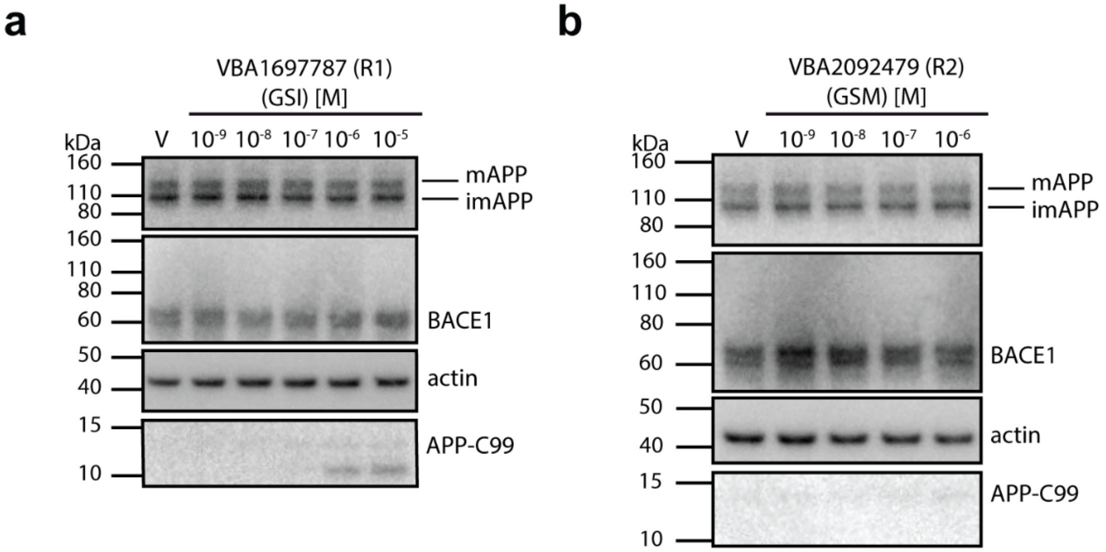

Supporting Information 4. Effect of VBA1697787 (R1) GSI and VBA2092479 (R2) GSM on protein levels in <u>stably APP-C99 overexpressing SH-SY5Y cells</u>. APP-C99-SH-SY5Y cells were treated with vehicle (V) or varying concentrations of the the VBA1697787 (GSI, R1) (a) and VBA2092479 (GSM, R2) (b).

a – b. Western blotting (representative of 3 independent experiments) of cell lysates for the detection of APP (*upper band*, mature; *lower band*, immature), BACE1, APP-C99 and actin (control).
