# Supplementary material for: Inhibition of BACE1 affected both its Aβ producing and degrading activities and increased Aβ42 and Aβ40 levels at high-level BACE1 expression
Source: J Biol Chem. 2024 Jun 27;300(8):107510. doi: 10.1016/j.jbc.2024.107510 (PMC11324814; doi:10.1016/j.jbc.2024.107510)
Supplement: Supporting Table 1 [file mmc5.docx]

| WT-SH-SY5Y | | | | | | |
| --- | --- | --- | --- | --- | --- | --- |
| *[R3] (M)* | **Aβ34** | | **Aβ40** | | **Aβ42** | |
|  | **M** | **SD** | **M** | **SD** | **M** | **SD** |
| *V* | 14.79 | 1.58 | 242.66 | 40.35 | 47.72 | 7.12 |
| *10^-9^* | 14.12 | 1.02 | 226.42 | 31.28 | 42.23 | 4.60 |
| *10^-8^* | 10.91 | 0.96 | 163.01 | 26.05 | 31.73 | 4.38 |
| *10^-7^* | 7.96 | 0.48 | 76.84 | 10.29 | 16.62 | 1.52 |
| *10^-6^* | 7.93 | 0.80 | 52.39 | 7.12 | 12.95 | 1.11 |
| *10^-5^* | 8.51 | 1.41 | 50.03 | 6.68 | 13.78 | 1.69 |
|  | | | | | | |
| BACE1-SH-SY5Y | | | | | | |
| *[R3] (M)* | **Aβ34** | | **Aβ40** | | **Aβ42** | |
|  | **M** | **SD** | **M** | **SD** | **M** | **SD** |
| *V* | 496.23 | 41.72 | 439.97 | 22.37 | 211.46 | 29.99 |
| *10^-9^* | 511.97 | 21.21 | 538.73 | 26.22 | 214.31 | 38.99 |
| *10^-8^* | 587.96 | 57.69 | 715.38 | 26.23 | 330.80 | 22.26 |
| *10^-7^* | 385.17 | 56.71 | 942.87 | 63.82 | 448.64 | 53.98 |
| *10^-6^* | 37.39 | 6.96 | 615.06 | 80.67 | 269.56 | 56.22 |
| *10^-5^* | 7.18 | 0.40 | 190.38 | 40.90 | 117.88 | 46.32 |
|  | | | | | | |
| *[N6] (M)* | **Aβ34** | | **Aβ40** | | **Aβ42** | |
|  | **M** | **SD** | **M** | **SD** | **M** | **SD** |
| *V* | 377.77 | 63.95 | 262.64 | 15.40 | 186.58 | 20.99 |
| *10^-9^* | 409.95 | 63.24 | 321.17 | 21.29 | 230.44 | 35.44 |
| *10^-8^* | 652.37 | 175.97 | 684.12 | 14.87 | 420.76 | 12.56 |
| *10^-7^* | 87.49 | 25.25 | 758.80 | 60.90 | 376.18 | 29.21 |
| *10^-6^* | 28.89 | 22.39 | 282.44 | 53.71 | 148.44 | 12.09 |
|  | | | | | | |
| *[N8] (M)* | **Aβ34** | | **Aβ40** | | **Aβ42** | |
|  | **M** | **SD** | **M** | **SD** | **M** | **SD** |
| *V* | 538.13 | 217.76 | 349.87 | 72.61 | 244.36 | 42.94 |
| *10^-9^* | 581.12 | 198.54 | 355.75 | 83.39 | 255.49 | 77.86 |
| *10^-8^* | 711.65 | 235.85 | 484.34 | 64.46 | 343.56 | 82.07 |
| *10^-7^* | 314.40 | 119.06 | 867.00 | 156.45 | 499.63 | 98.82 |
| *10^-6^* | 79.48 | 89.56 | 546.30 | 109.77 | 308.38 | 51.73 |
| *10^-5^* | 11.97 | 10.82 | 131.94 | 10.07 | 181.57 | 66.69 |
|  | | | | | | |
| *[N10] (M)* | **Aβ34** | | **Aβ40** | | **Aβ42** | |
|  | **M** | **SD** | **M** | **SD** | **M** | **SD** |
| *V* | 562.71 | 185.12 | 365.98 | 75.85 | 290.71 | 27.34 |
| *10^-9^* | 622.54 | 181.87 | 378.31 | 89.90 | 268.04 | 29.34 |
| *10^-8^* | 834.78 | 388.40 | 860.68 | 226.07 | 383.49 | 18.03 |
| *10^-7^* | 131.26 | 35.74 | 821.34 | 218.92 | 335.75 | 30.70 |
|  | | | | | | |
| *[N11] (M)* | **Aβ34** | | **Aβ40** | | **Aβ42** | |
|  | **M** | **SD** | **M** | **SD** | **M** | **SD** |
| *V* | 740.58 | 232.91 | 424.60 | 91.53 | 317.16 | 77.84 |
| *10^-9^* | 778.46 | 210.26 | 435.77 | 116.63 | 323.75 | 65.66 |
| *10^-8^* | 1076.07 | 309.50 | 883.72 | 81.11 | 424.38 | 10.51 |
| *10^-7^* | 436.26 | 121.99 | 1139.70 | 117.72 | 448.25 | 12.06 |
| *10^-6^* | 73.75 | 87.15 | 592.42 | 195.27 | 340.87 | 19.98 |
|  | | | | | | |
| APP-C99-SH-SY5Y | | | | | | |
| *[R3] (M)* | **Aβ34** | | **Aβ40** | | **Aβ42** | |
|  | **M** | **SD** | **M** | **SD** | **M** | **SD** |
| *V* | 272.27 | 26.89 | 17435.45 | 4011.28 | 6931.88 | 1119.14 |
| *10^-9^* | 262.34 | 32.13 | 15395.01 | 1490.04 | 6309.64 | 921.60 |
| *10^-8^* | 247.42 | 19.92 | 15840.08 | 2360.62 | 7271.80 | 639.38 |
| *10^-7^* | 198.95 | 17.95 | 17385.14 | 3193.77 | 6839.47 | 1404.54 |
| *10^-6^* | 172.70 | 11.39 | 18464.89 | 2464.08 | 7205.00 | 168.78 |
| *10^-5^* | 134.14 | 11.52 | 13513.45 | 1430.34 | 5711.14 | 718.30 |
|  | | | | | | |
| *[GSI] (M)* | **Aβ34** | | **Aβ40** | | **Aβ42** | |
|  | **M** | **SD** | **M** | **SD** | **M** | **SD** |
| *V* | 259.27 | 15.10 | 15273.63 | 1546.69 | 6617.67 | 1271.56 |
| *10^-9^* | 228.79 | 23.17 | 13032.53 | 1875.39 | 5145.07 | 2477.17 |
| *10^-8^* | 197.42 | 9.89 | 9859.99 | 541.79 | 4248.19 | 1774.24 |
| *10^-7^* | 99.05 | 4.83 | 3231.50 | 476.79 | 772.47 | 15.96 |
| *10^-6^* | 19.89 | 0.62 | 86.97 | 11.35 |  |  |
| *10^-5^* | 16.19 | 1.39 |  |  |  |  |
|  | | | | | | |
| *[GSM] (M)* | **Aβ34** | | **Aβ40** | | **Aβ42** | |
|  | **M** | **SD** | **M** | **SD** | **M** | **SD** |
| *V* | 317.15 | 27.37 | 15089.93 | 2963.29 | 5986.13 | 1462.86 |
| *10^-9^* | 326.27 | 35.33 | 13843.64 | 2121.47 | 5388.37 | 810.68 |
| *10^-8^* | 338.29 | 24.26 | 11491.02 | 1195.56 | 3256.19 | 563.57 |
| *10^-7^* | 356.13 | 41.25 | 3373.64 | 370.16 | 425.38 | 48.27 |
| *10^-6^* | 368.53 | 49.50 | 416.71 | 20.96 | 128.15 | 35.00 |

**Supporting Table 1. Mean and standard deviation of raw data from inhibitor treatments.** Mean and standard deviation are calculated for the absolute amounts of Aβ that are stated in Fig. 1f, 2f, 3a, 3c, 3e, 3g, 4f, 5a and 5e. M = mean, SD = sample standard deviation.
